# Large artery stiffening and mortality in a rat model of early vascular remodeling induced by intrauterine growth restriction and a high‐fat diet

**DOI:** 10.14814/phy2.15518

**Published:** 2022-12-02

**Authors:** Anastasiya Mankouski, Thomas A. Miller, R. Blair Dodson, Baifeng Yu, Yueqin Yang, Jingtong Liu, Daniel R. Machin, Anthony J. Donato, Robert A. McKnight, Erin K. Zinkhan

**Affiliations:** ^1^ Department of Pediatrics University of Utah Salt Lake City Utah USA; ^2^ Division of Pediatric Cardiology Maine Medical Center Portland Maine USA; ^3^ Departments of Surgery and Bioengineering The Pediatric Heart Lung Center and the Laboratory for Fetal and Regenerative Biology and the University of Colorado at Denver Anschutz Medical Campus Aurora Colorado USA; ^4^ Internal Medicine University of Utah Salt Lake City Utah USA; ^5^ GRECC VA Medical Center Salt Lake City Utah USA; ^6^ Florida State University Department of Nutrition and Integrative Physiology Tallahassee Florida USA; ^7^ Nutrition and Integrative Physiology University of Utah Salt Lake City Utah USA; ^8^ Biochemistry University of Utah Salt Lake City Utah USA

## Abstract

Intrauterine growth restriction (IUGR) and exposure to a high‐fat diet (HFD) independently increase the risk of cardiovascular disease (CVD) and hyperlipidemia. In our previous studies, IUGR increased blood pressure and promoted vascular remodeling and stiffness in early life, a finding that persisted and was augmented by a maternal HFD through postnatal day (PND) 60. The impact of these findings with aging and the development of hyperlipidemia and atherosclerosis remain unknown. We hypothesized that the previously noted impact of IUGR on hypertension, vascular remodeling, and hyperlipidemia would persist. Adult female rats were fed either a regular diet (RD) or high fat diet (HFD) prior to conception through lactation. IUGR was induced by uterine artery ligation. Offspring were weaned to either RD or HFD through PND 365. For both control (C) and IUGR (I) and rats, this resulted in the following six groups per sex: offspring from RD dams weaned to an RD (CRR and IRR), or offspring from HFD dams weaned to either an RD (CHR and IHR) or to an HFD (CHH and IHH). IHH male and female rats had increased large artery stiffness, a suggestion of fatty streaks in the aorta, and persistent decreased elastin and increased collagen in the aorta and carotid arteries. Post‐weaning HFD intake increased blood lipids regardless of IUGR status. IUGR increased HFD‐induced mortality. We speculate that HFD‐induced risk of CVD and mortality is potentiated by developmental programming of the ECM.

## Graphical abstract

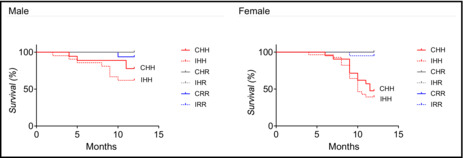

## 1 INTRODUCTION

Cardiovascular disease (CVD) remains a significant public health concern and a major source of morbidity and mortality (Roth et al., 2015). Public health efforts primarily focus on traditional risk factors for CVD, such as biological sex and consumption of a high‐fat diet. More recently, the role of the perinatal environment in programming life‐long vascular health and disease has been recognized (Barker, 2006; Barker, Osmond, et al., 1989a). The interplay between traditional cardiovascular risk factors and risk modification induced by the perinatal environment is not well understood.

Intrauterine growth restriction (IUGR) is often the consequence of an adverse perinatal environment such as placental insufficiency. IUGR impacts approximately 4%–8% of the population and increases the risk of multiple cardiometabolic diseases, including hypertension, hypercholesterolemia, CVD, and ischemic heart disease (Barker et al., 1992, 1993; Barker, Osmond, et al., 1989a; Barker, Winter, et al., 1989b). IUGR increases cholesterol in both human and animal models (Barker et al., 1993; Kannel et al., 1964; Zinkhan et al., 2014). Hypercholesterolemia has long been associated with and causes the development of atherosclerosis and CVD in a graded fashion (Kannel et al., 1964; Lozano et al., 2012; Spinler et al., 2003; Stamler et al., 1986). Therefore, identification of risk factors beyond the traditional risk factors and the mechanisms through which each of them increase CVD allows for the development of intervention to decrease morbidity and mortality associated with CVD.

In our previous studies using a rat model of IUGR combined with a maternal and post‐weaning high‐fat diet, we demonstrate the importance of developmental programming in the evolution of hypertension, hyperlipidemia, and changes to the extracellular matrix (ECM) early in life (Dodson et al., 2017; Miller et al., 2019; Zinkhan et al., 2016). This study aims to evaluate the persistence of these early‐life changes with aging in 12‐month‐old rats. Further, we aim to evaluate the impact of the perinatal environment on hypercholesterolemia and the potential development of fatty streaks. Last, we aim to evaluate large artery stiffness and ECM composition by evaluating both the aorta and carotid arteries. We hypothesize that the impact of the perinatal environment on hyperlipidemia and large vessel physiology would persist in 12‐month‐old rats and result in the development of fatty streaks, and that increases in ECM collagen thickness and alterations in elastic band composition would be vessel specific. To test our hypothesis, we analyzed aorta and carotid arteries of 12‐month‐old IUGR or normally grown male and female rats exposed to a maternal regular or high‐fat diet and who were weaned to a regular or high‐fat diet for vascular stiffness, ECM changes, serum lipids, and fatty streaks.

### 1.1 New and noteworthy

We report that intrauterine growth restriction impacts on early vascular aging persists and induces fatty streaks in the systemic vasculature in a rat model of maternal and offspring high‐fat diet consumption. Our study shows the importance of maternal and early life intervention to modify cardiovascular risk throughout life.

## 2 METHODS

### 2.1 Rat husbandry and study design

The University of Utah Animal Care and Use Committee approved all animal procedures. The rat model of IUGR and an HFD used in these studies was as previously described (Dodson et al., 2017; Miller et al., 2019; Zinkhan et al., 2016). In brief, fifty‐day‐old male and female Sprague Dawley CD rats were obtained from Charles River Laboratories, Inc (Wilmington, MA). These rats were exposed to 12‐h light–dark cycles per standard animal housing facility care. Adult male rats intended to be used for mating were kept on a regular rat chow from Harlan‐Teklad (TD.8640, Madison, WI). Before mating, female rats were fed either a regular rat chow (TD.8640) or a high‐fat diet rat chow (TD.110526). The regular rat chow (regular diet (RD)) contained 54% kcals carbohydrate, 29% kcals from protein, and 17% kcals from fat. The RD fat source was soybean oil at 60 g/kg food and contained 0.03% wt/wt cholesterol. The composition of the HFD was 40% kcals from carbohydrate, 16% kcals from protein, and 44% kcals from fat. The HFD contained a total of 65% saturated fat from a combination of soybean oil at 10 g/kg and milk fat, 1% wt/wt cholesterol, and 0.5% cholic acid to aid in fat absorption. The higher fat content of the HFD was achieved by decreasing both carbohydrate and protein content compared with the RD. The protein content in the HFD was chosen to mimic typical protein consumption in the United States (Wright et al., 2003) and does not cause growth restriction (Energy and protein requirements, 1973). Further, the protein content in the HFD is greater than the protein content used in low dietary protein studies (Boujendar et al., 2002).

No difference was found in the pre‐pregnancy maternal body weights after 5 weeks of dietary intervention. After 5 weeks of dietary intervention for the female rats, males were placed into the females' cages overnight for mating. Pregnancy day 0.5 was noted by the presence of sperm from a vaginal swab visualized under the microscope. Bilateral uterine artery ligation was performed on embryonic day 19.5 (E19.5) of an anticipated 21.5‐day gestation to induce IUGR as described previously (Fu et al., 2006). Anesthesia alone was used as a control to account for maternal medication exposure and because sham surgery has been shown to induce mild growth restriction in the offspring (Ogata et al., 1986).

**FIGURE 1:**
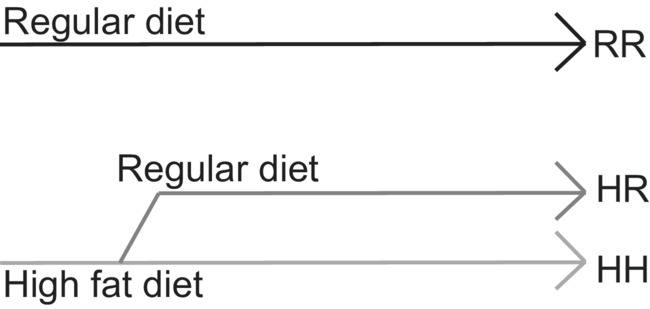
Schematic of study design. Our study design resulted in six study groups per sex: control (C) and IUGR (I) rats from dams fed a regular diet (RD) and weaned to RD (CRR and IRR), control and IUGR rats from dams fed a high fat diet (HFD) and weaned to RD (CHR and IHR), and control and IUGR rats from dams fed HFD and weaned to HFD (CHH and IHH). The top black line represents rats fed a RD throughout life (CRR and IRR rats), the middle gray line represents rats fed an HFD and weaned to a RD (CHR and IHR rats), and the bottom light gray line represents rats fed an HFD throughout life (CHH and IHH rats)

Pregnant female rats were allowed to deliver vaginally, offspring were weighed, and litters were culled to 6 at birth for rearing consistency. Pups remained with their own dam and the dam continued on the pre‐pregnancy diet through offspring postnatal day (PND) 21. At PND 21, offspring were separated from the dam and weaned either to the RD or the HFD described above. For all offspring this resulted in 3 diet groups and overall 6 different treatment groups: for both control (C) and IUGR (I) rats, the 3 diet groups included offspring from dams fed a RD and weaned to a RD (CRR and IRR), offspring from dams fed an HFD and weaned to a RD (CHR and IHR), and offspring from dams fed an HFD and weaned to an HFD (CHH and IHH) (Figure 1). Rats continued on their postnatal diet until 1 year of life at which time they were anesthetized with 8 mg/kg xylazine and 40 mg/kg ketamine prior to decapitation for tissue analysis. Only unrelated rats were used in these analyses, defined as one male and one female offspring rat per litter. For biomechanical analysis, the left carotid arteries were stored in 10% formalin overnight for histological analysis. For biochemical analysis, the right carotid arteries were frozen in liquid nitrogen. The descending thoracic aorta above the diaphragm was frozen in liquid nitrogen for biochemical analysis. A 5 mm segment of the abdominal aorta just below the diaphragm was fixed in 10% formalin overnight for histological analysis. The remainder of the abdominal aorta to the bifurcation of the iliac arteries were stored in cold calcium‐free PBS for oil red O staining.

### 2.2 Blood pressure analysis

Both systolic blood pressure (SBP) and diastolic blood pressure (DBP) were measured in unanesthetized rats via indirect tail cuff method (Blood pressure analysis system model MC4000, Hatteras Instruments, Cary, NC) according to manufacturer's instructions and as previously described (Dodson et al., 2017). Training rats on the blood pressure system began at 11 months of age and was continued daily by one operator until acclimated to the restraint and tail cuff system as defined by less than 10% variability in blood pressures during the blood pressure recording phase described below. Blood pressures were recorded just prior to PND 365 in non‐fasting rats. Each day of blood pressure training and testing was undertaken with 10 blood pressure measurements to reacclimatize the rat to the blood pressure monitoring system followed by 10 recorded blood pressure measurements. All blood pressure measurements were performed in a quiet room on a warmed platform and rats were handled by one person trained on the blood pressure monitoring system. An *n* = 6–14 unrelated rats per diet, surgical intervention, and sex were trained on the blood pressure system. All blood pressure analyses were performed by one person who was blinded to the rat group.

### 2.3 Aortic stiffness

Aortic stiffness was determined in vivo by measuring aortic pulse wave velocity (PWV) (Machin et al., 2020). Rats were anesthetized with 3% isoflurane and received 100% oxygen at 2 L/min flow rate via nose cone during testing. Rats were placed in the supine position on a heated platform. Blood velocity waveforms were acquired simultaneously at the transverse aortic arch and abdominal aorta with two 20‐MHz Doppler probes (Indus Instruments) and recorded using WinDAQ Pro+ software (DataQ Instruments). A precise measurement of the traveled distance between the Doppler probes was recorded using a scientific caliper. The transit time between Doppler sites was determined using the foot‐to‐foot method with WinDAQ Waveform Browser (DataQ Instruments). Aortic PWV was calculated as the traveled distance divided by the transit time. An *n* = 6–12 rats unrelated rats per diet, surgical intervention, and sex underwent testing, and due to poor imaging windows, results were obtained for *n* = 2–6 rats per group. Aortic stiffness was evaluated by one person who was blinded to the rat group.

### 2.4 Echocardiography

Cardiac function was assessed in vivo by echocardiography at 11.5 months of age using an MS 400 transducer and Vevo 2100 System (VisualSonics, FujiFilm) as previously described (Dodson et al., 2017). Briefly, prior to imaging, anesthesia was induced using 4% isoflurane in an induction chamber for 3 min (World Precision Instruments). Anesthesia was maintained with 2% isoflurane via nose cone during the echocardiogram and titrated to respiratory effort. A 2D image of the left ventricle in short axis at the level of the papillary muscles was obtained from the parasternal plane. M‐mode evaluation of fractional shortening was obtained in triplicate. An *n* = 10–19 unrelated rats per diet, surgical intervention, and sex underwent echocardiograms, and results were obtained for *n* = 6–18 rats per group. Echocardiography was performed and results analyzed by one person who was blinded to the rat group.

### 2.5 Aorta Oil Red O staining and serum lipids

Mixed arterial and venous blood was collected at the time of necropsy in serum separator tubes (BD Vacutainer, BD). A minimum of 1 ml blood was obtained from each rat for lipid and liver enzyme analysis. Blood was allowed to clot at room temperature for 30 minutes before centrifugal serum separation. Aliquots of serum were frozen at ‐80°C immediately after centrifugal serum separation, kept frozen until analysis, and analyzed within 1 week of collection. Standard serum lipid panels, aspartate aminotransferase (AST), and alanine aminotransferase (ALT) were analyzed at a clinical laboratory (ARUP laboratories). Serum was analyzed from 6–14 offspring per sex per group.

Abdominal adipose tissue was dissected from the abdominal aorta. The abdominal aorta was stained *en face* for 7 min with Oil Red O (Sigma‐Aldrich) and rinsed twice for 7 min each time in isopropanol (Sigma‐Aldrich). A ruler was placed next to the aorta and photographs were taken. The pictures of the stained aortas were analyzed with Bioquant True Color Windows Image Analysis System (R&M Biometrics) for the number of areas stained with Oil Red O and for the percent of the abdominal aorta stained with Oil Red O. Aortas were analyzed from 7–10 offspring per sex per group. Analysis of stained aortas was performed by two people who were blinded to the rat sex and group.

### 2.6 Histomorphometrics

Formalin‐fixed, paraffin‐embedded, sequential 5 μm sections of abdominal aorta or carotid artery were stained with either Masson's trichrome to visualize collagen fibers or Hart's stain to visualize elastic fibers as previously described (Dodson et al., 2017). In Masson's trichrome stains, collagen thickness in the adventitia perpendicular to the aortic wall was quantified using Bioquant True Color Windows Image Analysis System (R&M Biometrics) as previously described (Dodson et al., 2017). In Hart's elastic stains, the number of elastin fibers, and the thickness of the elastin fibers perpendicular to the lumen were quantified using Bioquant (R&M Biometrics) as previously described (Dodson et al., 2017). Analysis for both Masson's trichrome and Hart's stain were performed using eight measurements per high‐powered field and five high‐powered fields per vessel per rat. Unbiased analysis of elastin fiber density also was performed using Zeiss AxioImager M2 microscope (20x objective lens) with Stereo Investigator® software (MBF Bioscience, VT), as described below. Immunohistochemical measurements from *n* = 4–6 unrelated rats per diet, surgical intervention, and sex were used for analyses in this study, and these aorta and carotid sections were used for stereology analysis. Staining, imaging, and analysis of vessel histomorphometrics were performed by one person who was blinded to the rat sex and group.

### 2.7 Stereology

Aorta and carotid artery sections was imaged using Zeiss AxioImager M2 microscope (20x objective lens) with Stereo Investigator® software (MBF Bioscience). To estimate the percentage of thin and large elastin within aorta and carotid artery wall, the Area Fraction Fractionator® probe was used. Uniform random sampling of the tissue was performed according to standard stereological principles, where sampling parameters (for example, grid size and counting frame size) were empirically determined to arrive at low coefficients of error (CE). CE is defined as the standard error of the mean of repeated estimates divided by the mean and used as a measure of the accuracy of the stereological procedure. The probe grid size and counting frame size were empirically determined to yield average cumulative error values <0.1. Three different types of markers were placed on points over large elastin, thin elastin, or no elastin manually. Quantification of aorta and carotid artery wall elastin was done by image analysis using coded masked images. The estimate of the percentage of large or thin elastin by either aorta or carotid artery wall area were calculated. Stereology measurements from *n* = 4 unrelated rats per diet, surgical intervention, and sex were used for analyses in this study, and these aorta sections were used for immunohistochemical analysis. Stereology measurements were performed by two people who were blinded to the rat sex and group.

### 2.8 Aorta protein quantification

Flash frozen thoracic aorta and right carotid artery protein was isolated using whole cell lysates in buffer (150 mM NaCl, 50 mM Tris pH 7.4, 1 mM EDTA, 0.25% Na‐deoxycholate, 1% Igepal CA‐630). Bicinchoninic acid assay with bovine serum albumin as a standard curve was used to measure protein concentrations. Fifty micrograms protein per sample was analyzed on a 24‐well 10% SDS PAGE gel (Bio‐Rad). The protein was transferred to a polyvinylidene difluoride membrane and blocked with 5% milk. Hypoxanthine phosphoribosyltransferase 1 (Hprt1) (Proteintech) was used as a loading control because Hprt1 did not differ by diet or intrauterine environmental. Antibodies used in this study included Elastin (Abcam), Lysyl oxidase (Lox) (Novus Biologicals), Matrix metalloproteinase 2 (MMP‐2) (Santa Cruz Biotechnology), MMP‐9 (Abcam), Tissue inhibitor of metalloproteinases 2 (TIMP‐2) (Santa Cruz Biotechnology), Advanced glycosylation end product specific receptor (AGER, also known as RAGE) (Novus), and Advanced glycosylation end products (AGE) (GeneTex). Protein was visualized using Western Lightning enhanced chemiluminescence (PerkinElmer Life Sciences) with goat anti‐rabbit horseradish peroxidase secondary antibody from Cell Signaling Technology. Western blot images were analyzed using a Kodak Image Station 2000R (Eastman Kodak/SIS). Aortic protein from 6 unrelated rats per diet, surgical intervention, and sex were used for analyses in this study, and these rats also were used for histochemical.

Because our study design resulted in a total of six diet and intrauterine condition groups, the large sample size precluded running all six samples per group on a single western blot gel. To address this challenge, sexes were run on separate gels and analyzed separately, and no statistical comparisons were made between sexes, as previously described (Miller et al., 2019). Within each sex, protein samples from all six CRR and IRR rats were run on every gel along with either all six samples from both CHR and IHR or both CHH and IHH rats. Each target protein was first compared to the loading control (Hprt1). For the graphical representation of data, protein levels from the CRR rats were compared between the two western blot gels (one gel containing CRR, IRR, CHR, and IHR protein samples, and other gel containing CRR, IRR, CHH, and IHH protein samples). For purposes of graphical representation, protein levels from the six CHR and IHR rats were normalized for differences between the CRR results between the gels to account for differences in baseline loading and gel exposure. Because CHR, IHR, CHH, and IHH protein samples were not run on the same gel, no analysis was conducted comparing CHR and IHR data to CHH and IHH data. Analysis of CHR and IHR data was completed only relative to the CRR samples run on the same gel, and analysis of CHH and IHH data was completed only relative to the CRR samples run on the same gel. For concise data representation in the figures, a separate gel was run with one random sample from each group and included in the figure as a representative blot, but no data analysis was performed on the representative blots.

### 2.9 Aorta collagen quantification

Descending thoracic aorta was flash frozen in liquid nitrogen and stored at ‐80°C until analysis. Frozen thoracic aorta was crushed over liquid nitrogen. Between 20–30 mg frozen crushed aorta tissue was weighed and weights recorded. Soluble and insoluble collagen fractions were separated with a solution of 0.1 mg/ml pepsin in 0.5 M acetic acid overnight on ice. Soluble collagen was isolated, concentrated, and measured according to the manufacturer's instructions (Sircol Soluble Collagen Assay, Biocolor). Insoluble collagen was measured without first concentrating the samples according to manufacturer's instructions (Sircol Insoluble Collagen Assay, Biocolor). Data were expressed as μg total collagen per mg initial aorta tissue weight. Aortic protein from 6 unrelated rats per diet, surgical intervention, and sex were used for analyses in this study, and tissues from these rats were also used in oil red O analysis. Due to the smaller size of carotid artery tissue, this analysis was not able to be done on carotid arteries. Collagen quantification was performed by one person who was blinded to the rat sex and group.

### 2.10 Statistics

Survival curves were analyzed using Log‐rank (Mantel‐Cox test). Other data were expressed as mean +/− standard deviation (SD). The statistic package GraphPad Prism version 8.4.3 (GraphPad Software) was used for analyses. Non‐parametric statistics (Kruskal–Wallis followed by Dunn's multiple comparison test) was used for data analysis, with the following comparison groups: all groups to CRR; IHR to CHR; and IHH to CHH. A *p* value of ≤ 0.05 was considered to be statistically significant.

## 3 RESULTS

### 3.1 Survival curves and analysis of rats that died before the study end point

**FIGURE 2:**
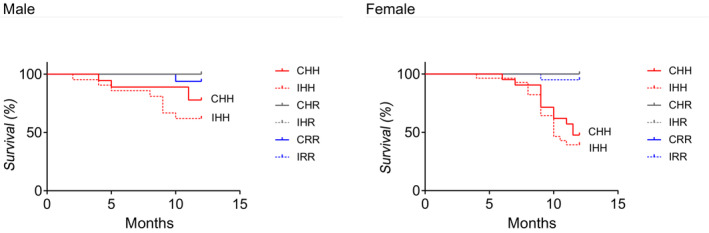
Survival curves. CHH and IHH male and female rats had increased mortality compared to all other sex‐matched rat groups (Log‐rank (Mantel‐Cox test)). Data from the following number of rats per group were included: male CRR 16, male IRR 15, male CHR 15, male IHR 15, male CHH 21, male IHH 29, female CRR 19, female IRR 20, female CHR 18, female IHR 19, female CHH 22, female IHH 28. Data are shown as follows: CRR in blue solid lines, IRR in blue dotted lines, CHR in gray solid lines, IHR in gray dotted lines, CHH in red solid lines, and IHH in red dotted lines

At 12 months, decreased survival rates were seen in male CHH and male IHH rats (*p* = 0.005 Log‐rank (Mantel‐Cox test)), and female CHH and female IHH rats (*p* < 0.001 Log‐rank (Mantel‐Cox test)) (Figure 2). All other groups had at least 95% survival at 12 months. The median survival for female CHH rats was 11.5 months, and for female IHH rats was 10 months. All other rat groups had a median survival of at least 12 months. The University of Utah Animal Care and Use Committee was notified of the unanticipated premature deaths, and after an investigation, allowed the study to continue.

Of the rats that died before 12 months of life, necropsy was performed on approximately half of the rats, all of which were performed within 12 h of death, as determined by the rat being alive and subjectively well appearing within the 12 h prior to death. Three rats were found to have rapid labored breathing and cyanotic mucosa and were humanely euthanized prior to necropsy. None of the rats who died prematurely had an obvious cause of death noted on necropsy other than two additional rats that were humanely euthanized, one CRR male rat who had a large tumorous growth over the left hip and one IRR female rat that had overgrown front teeth. Of the rats that died before 12 months of age and who underwent necropsy, mean heart mass to body mass ratios were as follows: CHH female rats 0.009 (*n* = 10), IHH female rats 0.01 (*n* = 7), IRR female rats 0.003 (*n* = 1), IHH male rats 0.006 (*n* = 4), and CRR male rats 0.005 (*n* = 1). Due to the small number of rats that died prematurely and that underwent necropsy, only female CHH and IHH female rats that died prematurely were compared to CHH and IHH female rats that survived to the study endpoint of 12 months, and male IHH rats that died prematurely to male IHH rats that survived. Both female CHH and IHH rats that died prematurely had increased heart mass to body mass ratios compared to those that survived (*p* < 0.001). Male IHH rats that died prematurely had increased heart mass to body mass ratios compared to male IHH rats that survived (*p* < 0.003, Mann Whitney test).

### 3.2 Blood pressure and aortic pulse wave velocity

**FIGURE 3:**
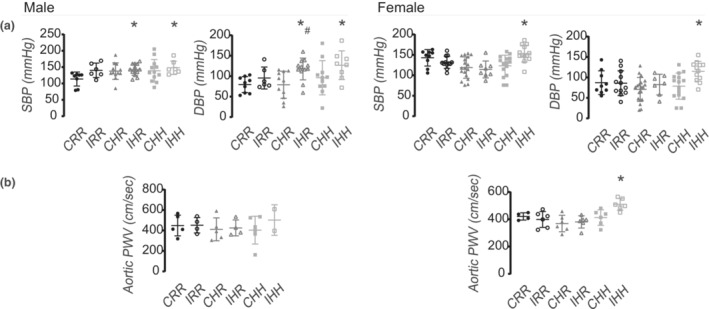
Blood pressure and aorta stiffness by PWV. (a) Systolic and diastolic blood pressure was increased in IHH and IHR male rats, and in IHH female rats. (b) Aortic PWV was increased in IHH female rats. Blood pressure data from the following number of rats per group were included: male CRR 8, male IRR 6, male CHR 12, male IHR 13, male CHH 12, male IHH 7, female CRR 9, female IRR 12, female CHR 18, female IHR 8, female CHH 15, female IHH 13. PWV data from the following number of rats per group were included: male CRR 5, male IRR 4, male CHR 5, male IHR 4, male CHH 6, male IHH 2, female CRR 4, female IRR 6, female CHR 6, female IHR 6, female CHH 6, female IHH 6. * indicates *p* < 0.05 compared to CRR rats, and # indicates *p* < 0.05 for IHR compared to CHR rats

Increased systolic and diastolic blood pressures were found in IHR and IHH male rats, and increased diastolic blood pressure in IHR male rats (Figure 3). IHH female rats were the only group that had increased systolic and diastolic blood pressure compared to CRR rats.

Consistent with blood pressure data, IHH female rats had increased aortic PWV compared to CRR rats (Figure 3). Due to the size and increased adiposity of the CHH and IHH male rats, few high‐quality PWV readings were obtained. Of the high‐quality data obtained, there were no differences in aortic PWV between any of the male rat groups.

### 3.3 Echocardiography and heart mass

**FIGURE 4:**
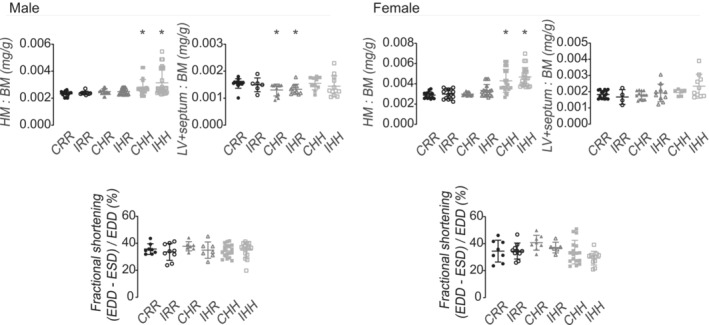
Echocardiography and heart mass. Left ventricle plus septal mass relative to body mass was decreased in CHR and IHR rats. No difference was seen between groups for fractional shortening by echocardiography. Echocardiography data from the following number of rats per group were included: male CRR 8, male IRR 9, male CHR 7, male IHR 7, male CHH 18, male IHH 16, female CRR 8, female IRR 10, female CHR 8, female IHR 6, female CHH 17, female IHH 11. Heart mass data from the following number of rats per group were included: male CRR 13, male IRR 9, male CHR 18, male IHR 20, male CHH 21, male IHH 29, female CRR 14, female IRR 15, female CHR 14, female IHR 18, female CHH 22, female IHH 23. Left ventricle and septal mass data from the following number of rats per group were included: male CRR 14, male IRR 6, male CHR 11, male IHR 11, male CHH 10, male IHH 12, female CRR 16, female IRR 4, female CHR 13, female IHR 10, female CHH 6, female IHH 10. * indicates *p* < 0.05 compared to CRR rats. Heart mass relative to body mass was increased in CHH and IHH male and female rats

CHH and IHH male and female rats had increased wet whole heart mass to body mass ratios compared to respective CRR rats (Figure 4). CHR and IHR male rats had decreased wet left ventricle plus septal mass to body mass ratios compared to CRR male rats (Figure 4).

There were no differences in fractional shortening (FS), LVIDd, IVSd, and LVPWd between any of the groups (FS shown in Figure 4). On echocardiography, two rats were noted to have significant bradycardia with heart rates approximately 80 beats per minute and noticeable pericardial effusions at the beginning of the study. These rats subsequently woke from anesthesia without difficulty but died within 24 h. Data from these two rats were not used in analyses in this study other than for necropsy data for rats that died prematurely.

### 3.4 Serum lipids and fatty streaks

**FIGURE 5:**
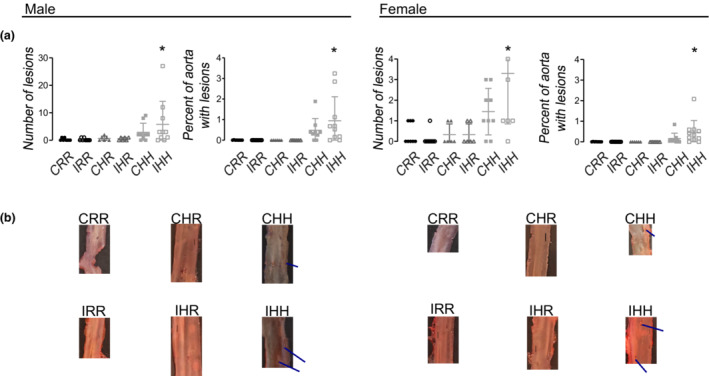
Aorta fatty streaks by oil red o staining. Number of oil red o stained lesions and percent of the aorta with oil red o staining was increased in male and female IHH rats. (a) Analysis of number of oil red o stained areas of the *en face* aorta and the percent of stained areas compared to the total *en face* aorta analyzed. (b) Representative images of *en face* stained abdominal aortas. Vertical black scale bars in each image represent 1 mm. Horizontal blue bars highlight areas in the aorta that have oil red o staining, excluding fatty tissue outside of the aorta. Oil red o staining data from the following number of rats per group were included: male CRR 8, male IRR 8, male CHR 7, male IHR 7, male CHH 9, male IHH 10, female CRR 8, female IRR 8, female CHR 7, female IHR 7, female CHH 9, female IHH 10. * indicates *p* < 0.05 compared to CRR rats

An increased number of Oil Red O‐stained lesions in the abdominal aorta and an increased percent of the abdominal aorta that stained with Oil Red O was seen in CHH and IHH male and female rats (Figure 5).

CHH and IHH male rats had increased total cholesterol and triglyceride levels compared to CRR rats (Supplemental Figure S1, https://figshare.com/s/1eca39bfc3bc910f173e). CHH male rats had increased low density lipoprotein cholesterol levels compared to CRR rats. CHH and IHH female rats had increased total cholesterol and triglyceride levels and decreased high density lipoprotein levels compared to CRR rats. CHH female rats had increased low density lipoprotein cholesterol levels compared to CRR rats. There were no statistically significant differences between groups for serum AST and ALT levels.

### 3.5 Aorta elastin and protein drivers of elastin remodeling

**FIGURE 6:**
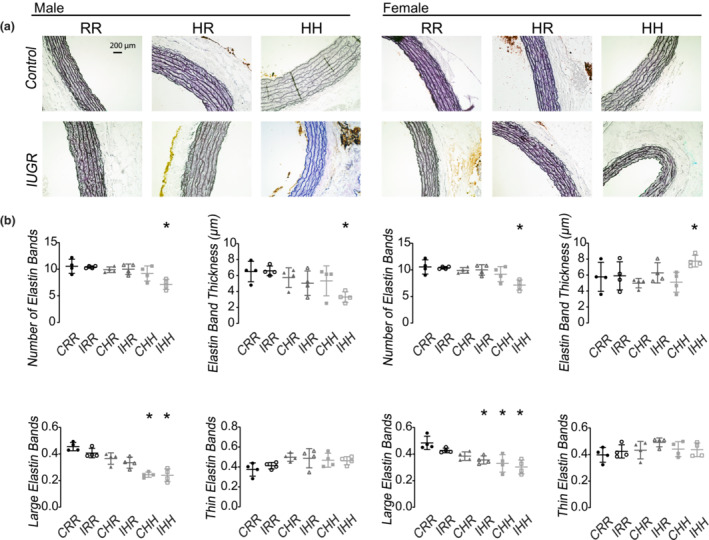
Aorta elastin immunohistochemistry. (a) Representative images of abdominal aortas stained for elastin. All images were taken at 63× magnification. Scale bar was displayed in the male CRR image and represented 200 μm. (b) IHH male and female rats had fewer aorta elastin bands and large elastin bands. IHH male rats had thinner aorta elastin bands, and IHH female rats had thicker aorta elastin bands. Data for 4 rats per sex, diet, and intrauterine condition were used for aorta immunohistochemistry analysis. * indicates *p* < 0.05 compared to CRR rats

IHH male rats had fewer and thinner elastin bands in the abdominal aorta compared to CRR rats (Figure 6). IHH female rats had fewer and thicker elastin bands in the abdominal aorta compared to CRR rats.

IHH male rats had increased thoracic aorta Lox protein levels compared to CRR rats (Supplemental Figure S2, https://figshare.com/s/1eca39bfc3bc910f173e). IHH female rats had increased thoracic aorta Lox protein compared to CHH female rats, and decreased thoracic aorta MMP‐2 protein compared to CRR female rats.

### 3.6 Aorta collagen and protein drivers of collagen remodeling

**FIGURE 7:**
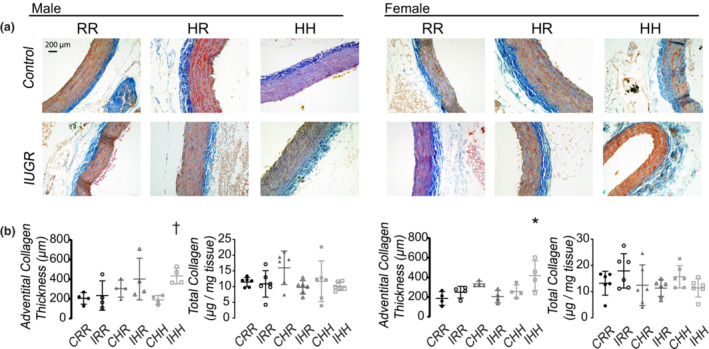
Aorta collagen immunohistochemistry. (a) Representative images of abdominal aortas stained for collagen. All images were taken at 63× magnification. Scale bar was displayed in the male CRR image and represented 200 μm. (b) IHH male and female rats had increased aorta adventitial collagen thickness. Data for 4 rats per sex, diet, and intrauterine condition were used for aorta immunohistochemistry analysis. * indicates *p* < 0.05 compared to CRR rats, and +indicates *p* < 0.05 for IHH rats compared to CHH rats

IHH female rats had increased adventitial collagen thickness in the abdominal aorta compared to CRR female rats (Figure 7).

IHH female rats had increased thoracic aorta advanced glycation end products compared to CRR rats (Supplemental Figure S3, https://figshare.com/s/1eca39bfc3bc910f173e)

### 3.7 Carotid elastin and protein drivers of elastin remodeling

**FIGURE 8:**
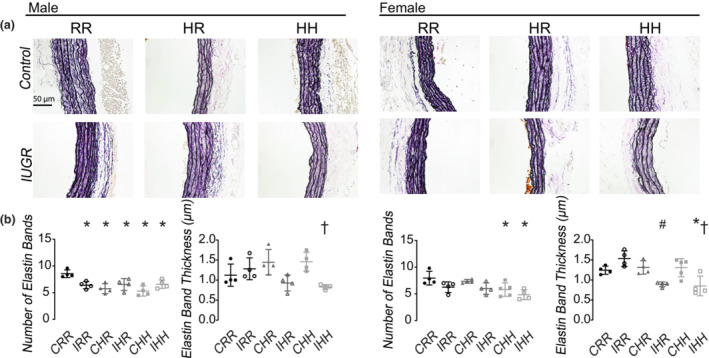
Carotid elastin immunohistochemistry. (a) Representative images of carotid arteries stained for elastin. All images were taken at 80x magnification. Scale bar was displayed in the male CRR image and represented 50 μm. (b) All male rat groups had fewer carotid elastin bands compared to CRR male rats. IRR male rats had thinner carotid elastin bands. Female CHH and IHH had fewer carotid elastin bands. Female IHH rats had thinner carotid elastin bands. Data for 6 rats per sex, diet, and intrauterine condition were used for carotid immunohistochemistry analysis. * indicates *p* < 0.05 compared to CRR rats, # indicates *p* < 0.05 for IHR rats compared to CHR rats, and + indicates *p* < 0.05 for IHH rats compared to CHH rats

All male rat groups had fewer carotid elastin bands compared to the CRR male rats (Figure 8). IHH male rats had thinner carotid elastin bands compared to CHH male rats. CHH and IHH female rats had fewer carotid elastin bands compared to CRR female rats. IHH female rats had thinner carotid elastin bands compared to both CRR and CHH female rats, and IHR female rats had thinner elastin bands compared to CRR female rats.

IHH male rats had decreased carotid TIMP‐2 protein compared to CHH male rats (Supplemental Figure S4, https://figshare.com/s/1eca39bfc3bc910f173e). IHH and IRR female rats had increased carotid MMP‐2 protein compared to CRR female rats.

### 3.8 Carotid collagen and protein drivers of collagen remodeling

**FIGURE 9:**
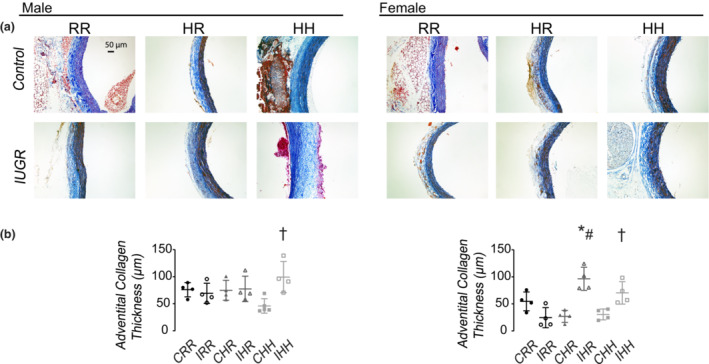
Carotid collagen immunohistochemistry. (a) Representative images of carotid arteries stained for collagen. All images were taken at 40× magnification. Scale bar was displayed in the male CRR image and represented 50 μm. (b) IHH male and female rats had thicker carotid adventitial collagen compared to CHH male and female rats, respectively. IHR male rats had thicker carotid adventitial collagen compared to CRR and CHR male rats. Data for 6 rats per sex, diet, and intrauterine condition were used for carotid immunohistochemistry analysis. * indicates *p* < 0.05 compared to CRR rats, # indicates *p* < 0.05 for IHR rats compared to CHR rats, and+ indicates *p* < 0.05 for IHH rats compared to CHH rats.

IHH male rats had increased carotid adventitial collagen thickness compared to CHH male rats (Figure 9). IHR and IHH female rats had increased carotid adventitial collagen thickness compared to CRR female rats, and IHR female rats had increased carotid adventitial collagen thickness compared to CHR female rats.

IHR female rats had increased carotid AGEs and RAGEs compared to CRR female rats (Supplemental Figure S5, https://figshare.com/s/1eca39bfc3bc910f173e).

## 4 DISCUSSION

The primary findings of this study are that IUGR persistently increases blood pressure and alters the vascular extracellular matrix in rats exposed to a high‐fat diet (Dodson et al., 2017; Miller et al., 2019). Further, these IHH rats have increased mortality and suggestions of fatty streaks that are not attributable to increased lipid levels alone. While specific vascular extracellular matrix findings are vessel specific, both aorta and carotid vasculature shows IUGR and high‐fat diet‐induced decreases in elastin band number and increases in adventitial collagen thickness. These findings suggest that developmental programming of the extracellular matrix potentiates diet‐induced cardiovascular disease throughout life.

At PND 21, our IUGR and maternal HFD rats exhibit both aorta extracellular matrix alterations and molecular markers of vascular remodeling (Dodson et al., 2017). However, consistent with our data presented at day 60, many of the molecular markers of vascular remodeling in the aorta remain normalized in rats at 1 year of age (Miller et al., 2019). While the carotid arteries were not histologically evaluated in our prior studies, the relative absence of molecular markers of vascular remodeling in the aorta and carotid arteries suggests that vascular remodeling in both arteries is not actively occurring at 1 year, but rather set on a trajectory by the perinatal environment that persists throughout life.

Elastin formation occurs over a limited period of time. Therefore, damaged or degraded elastin fibers are not repaired, but rather replaced with collagen fibers (Cocciolone et al., 2018). With aging, the human aorta elastin to collagen ratio decreases over time (Hosoda et al., 1984). With aging, decreased elastin reduces the distensibility of the aorta during systole and may injure the microvasculature (Cocciolone et al., 2018). Early vascular aging, noted by a decrease in the elastin to collagen ratio, may increase microvascular injury and predispose to end organ damage (O'Rourke & Safar, 2005). Our data have shown a persistent IUGR and HFD‐induced decrease in elastin content in the aorta from PND 21 through 1 year, and an increase in collagen content at PND 21 and 1 year. Our data may indicate that increased blood pressures seen by IHH male and female rats is induced by early vascular aging programmed by the perinatal environment.

Developmental programming of vascular changes is known to be vessel specific. Human placental tissue from growth restricted fetuses shows decreased collagen and increased elastin associated with umbilical vascular resistance and pulsatility indices (Saw et al., 2018). The aorta and carotid arteries of growth restricted sheep demonstrates altered collagen to elastin ratios and increased stiffness (Dodson et al., 2014; Dodson, Rozance, Fleenor, et al., 2013a; Dodson, Rozance, Reina‐Romo, et al., 2013b). Decreased mRNA of elastin and genes associated with elastin fiber formation is found in the lung of growth restricted rats (Joss‐Moore et al., 2011). Our study shows similar systemic vascular ECM changes of decreased elastin band number and thickness and increased adventitial collagen. Both systemic vessels investigated in this study show similar findings of early vascular aging.

The impact of maternal HFD consumption and IUGR is not fully reversible when the rats were weaned to a regular rat chow. The IHR male rats had persistently elevated systolic and diastolic blood pressure, less aorta elastin protein, and alteration in aorta elastin staining on immunohistochemistry (Miller et al., 2019). Early vascular aging as evidenced by decreased elastin content of the aorta may contribute to vascular stiffening and increased blood pressure in the IHR male rats. These findings suggest that hypertension associated with known dietary risk factors can be augmented by developmental programming even in the absence of ongoing diet exposure. Although no end‐organ damage was found in the IHR male rats by 12 months of age, intrauterine programming of vascular stiffening may predispose to end‐organ damage in older rats or in longer‐lived species even when consuming a standard diet.

Interestingly, the IHR female rat did not develop higher blood pressure, increased aorta PWV, or alterations in aorta collagen or elastin. These findings suggest that the 1‐year‐old IHR female rat is relatively protected from early vascular aging in our rat model. In humans, women born with low birth weight have increased blood pressure after menopause, as reviewed by Davis et al (Davis et al., 2017). The changing hormonal mileu during and after menopause may contribute to the enhanced susceptibility to hypertension in women born with low birth weight compared to women born with normal birth weight (Davis et al., 2017). While hormonal alterations were not evaluated in our study, female rats are not known to go through a defined menopause. However, with further aging through the rat lifespan of approximately two to two‐and‐a‐half years, increased susceptibility to hypertension may be seen in IHR female rats in our rat model.

The impact of biological sex on IUGR‐induced outcomes has long been of interest to investigators (Barker & Osmond, 1986; Barker, Osmond, et al., 1989a; Sandboge et al., 2012). Various hypotheses have been proposed for the disparate impact of intrauterine conditions and biological sex on physiological outcomes, including alterations in placental size and nutrient transfer, fetal programming of the sympathetic nervous system and hormonal regulation, structural changes in organ development, balance of apoptosis, and epigenetic phenomena (Herrera et al., 2014; Sandboge et al., 2012; van Abeelen et al., 2011). Male humans have relatively smaller placentas and are more dependent on maternal nutrition status than females, which may predispose males to hypertension in adulthood (Eriksson et al., 2010). Alterations in histone modifying proteins were associated with aorta and carotid stiffness in salt‐sensitive male rats (Herrera et al., 2014). While the underlying mechanism through which biological sex influenced IUGR‐induced alterations in our rats was beyond the scope of this study, we speculate that multiple mechanisms interact to lead to the ultimate changes in physiology and extracellular matrix changes.

Maternal overnutrition during pregnancy and lactation increases the risk of cardiovascular and cardiometabolic disorders in offspring and exacerbates the formation of atherosclerosis (Drake & Reynolds, 2010; Fleming et al., 2018; Godfrey et al., 2017; Reynolds et al., 2013; Wakana et al., 2015). In our study, while CHH and IHH offspring had increased serum lipid levels, increased fatty streaks were only seen in IHH male and female rats. Increased fatty streaks induced by the combination of IUGR and an HFD but not by an HFD alone suggests that other underlying pathways potentially exist for the deposition of lipid into the aorta in the setting of IUGR. In‐depth exploration of these potential underlying pathways was beyond the scope of this study. However, these potential pathways could include alteration of macrophage‐induced inflammation, calcification of the vascular smooth muscle cells, and alteration of the underlying extracellular matrix (Wakana et al., 2015).

Maternal obesity and overnutrition during pregnancy also increase offspring all‐cause mortality and hospital admission due to a cardiovascular event (Reynolds et al., 2013). In mice, maternal obesity induced offspring cardiac hypertrophy and long‐term alteration in carbohydrate and lipid metabolism, suggesting life‐long increased risk of cardiovascular disease (Vaughan et al., 2022). Further, maternal obesity and offspring cardiac dysfunction programmed during gestation are mechanistically linked through adiponectin (Vaughan et al., 2020). Maternal and offspring HFD consumption increases mortality in our rat model, a finding which is further potentiated by IUGR in both male and female offspring. While the cause of increased mortality in our rats remains unknown, and statistical analysis was not undertaken for the heart weights of the rats that underwent necropsy, the data suggest that rats that died prematurely may have had increased heart weight to body weight ratios relative to those that survived to 12 months. Further, two rats that died prematurely had bradycardia and pericardial effusions. These data suggest that the increased mortality in the HFD fed rats may have been of cardiac origin.

Several potential limitations exist in our current study. First, as with our previous studies at PND 21 and PND 60, this study evaluates one time point, at 1 year of age. Second, the HFD rat chow contains less protein than the regular rat chow. However, the amount of protein in the HFD rat chow is sufficient for normal rat growth and mimics the typical American diet (Energy and protein requirements, 1973; Wright et al., 2003). Third, a sham surgery group was not included in our study because sham surgery induces a mild growth restriction, therefore our control group consisted of an anesthesia control as is consistent with previous rat models of IUGR (Zinkhan et al., 2016). Fourth, tail‐cuff blood pressure measurements were used to approximate blood pressure, which may have underestimated the impact of the perinatal environment on blood pressure. Fifth, due to limited availability of tissues in a small animal model, tissues were evaluated from multiple locations along the aorta. Our previous work in a large animal model of IUGR demonstrates changes across the regions of the aorta varied subtly, but increased vascular stiffness and alteration of collagen and elastin was similar throughout (Dodson et al., 2014; Dodson, Rozance, Fleenor, et al., 2013a; Dodson, Rozance, Reina‐Romo, et al., 2013b). Sixth, the PWV studies in male IHH rats have a low N due to challenges obtaining data due to high mortality and high adiposity in this group. The data obtained are presented to enhance transparency of data reporting and parallel presentation of the data.

In summary, IUGR persistently increases blood pressure and alters the extracellular matrix of systemic blood vessels in high‐fat diet fed rats. These findings are not fully reversible by weaning to a regular rat chow. The perinatal environment‐induced changes to the extracellular matrix are set early in life and may predispose offspring to increased cardiovascular risk and mortality with aging.

## AUTHOR CONTRIBUTIONS

Anthony J. Donato: analyzed data, interpreted results, revised manuscript, approved finalized manuscript. Blair Dodson: designed the study, analyzed data, interpreted results, revised manuscript, approved finalized manuscript. Erin K. Zinkhan designed the study, performed experiments, analyzed data, interpreted results, drafted manuscript, revised manuscript, approved finalized manuscript. Jingtong Liu: performed experiments, analyzed data, interpreted results, revised manuscript, approved finalized manuscript. Daniel Machin: performed experiments, analyzed data, interpreted results, revised manuscript, approved finalized manuscript. Anastasiya Mankouski: designed the study, performed experiments, analyzed data, interpreted results, drafted manuscript, revised manuscript, approved finalized manuscript. Robert A McKnight: designed the study, interpreted results, revised manuscript, approved finalized manuscript. Thomas A. Miller: designed the study, analyzed data, interpreted results, revised manuscript, approved finalized manuscript. Yueqin Yang: performed experiments, analyzed data, revised manuscript, approved finalized manuscript. Baifeng Yu: performed experiments, analyzed data, interpreted results, revised manuscript, approved finalized manuscript

## Supporting information


Data S1.

Figure S1.

Figure S2.

Figure S3.

Figure S4.

Figure S5.
