# Supplementary material for: Large artery stiffening and mortality in a rat model of early vascular remodeling induced by intrauterine growth restriction and a high‐fat diet
Source: Physiol Rep. 2022 Dec 2;10(23):e15518. doi: 10.14814/phy2.15518 (PMC9718947; doi:10.14814/phy2.15518)
Supplement: Supplementary file 1 — Data S1. [file PHY2-10-e15518-s006.pdf]

A) Representative images of carotid arteries stained for collagen. All images were taken at 40x magnification. Scale bar was displayed in the male CRR image and represented 50 µm. B) IHH male and female rats had thicker carotid adventitial collagen compared to CHH male and female rats, respectively. IHR male rats had thicker carotid adventitial collagen compared to CRR and CHR male rats. Data for 6 rats per sex, diet, and intrauterine condition were used for carotid immunohistochemistry analysis. * indicates $p<0.05$ compared to CRR rats, # indicates $p<0.05$ for IHR rats compared to CHR rats, and + indicates $p<0.05$ for IHH rats compared to CHH rats.

For Review Only

Supplemental figure legends:

Supplemental figure 1: Fasting serum lipid levels.

CHH and IHH male and female rats had increased serum total cholesterol and triglyceride levels. Serum lipid data from the following number of rats per group were included: male CRR 6, male IRR 6, male CHR 6, male IHR 6, male CHH 12, male IHH 14, female CRR 6, female IRR 6, female CHR 6, female IHR 6, female CHH 12, female IHH 10. * indicates $p<0.05$ compared to CRR rats.

Supplemental figure 2: Expression of markers of vascular remodeling of elastin in the aorta.

A) Representative blot images were displayed above the graphical representation of each protein with kDa marker along the left side of each blot as marked. B) IHH male and female rats had increased LOX protein levels. Data for 6 rats per sex, diet, and intrauterine condition were used for protein level analysis. * indicates $p<0.05$ compared to CRR rats, and + indicates $p<0.05$ for IHH rats compared to CHH rats.

Supplemental figure 3: Expression of markers of oxidative stress and vascular remodeling of collagen in the aorta.

A) Representative blot images were displayed above the graphical representation of each protein with kDa marker along the left side of each blot as marked. B) Male IHR rats had less

AGE protein in the aorta. Female IHH rats had increased AGE protein in the aorta. Female IHR rats had increased RAGE protein in the aorta. Data for 6 rats per sex, diet, and intrauterine condition were used for protein level analysis. * indicates $p<0.05$ compared to CRR rats, and # indicates $p<0.05$ for IHR rats compared to CHR rats.

Supplemental figure 4: Expression of markers of vascular remodeling of elastin in the carotid artery.

A) Representative blot images were displayed above the graphical representation of each protein with kDa marker along the left side of each blot as marked. B) IHH male rats had decreased TIMP-2 protein. Female IRR and IHH rats had increased MMP-2 protein. Data for 6 rats per sex, diet, and intrauterine condition were used for protein level analysis. * indicates $p<0.05$ compared to CRR rats, and + indicates $p<0.05$ for IHH rats compared to CHH rats.

Supplemental figure 5: Expression of markers of oxidative stress and vascular remodeling of collagen in the carotid artery.

A) Representative blot images were displayed above the graphical representation of each protein with kDa marker along the left side of each blot as marked. B) IRR female rats had increased AGE and MMP-9 protein levels. IHR female rats had increased RAGE and MMP-9 protein levels. Data for 6 rats per sex, diet, and intrauterine condition were used for protein level analysis. * indicates $p<0.05$ compared to CRR rats

For Review Only
