# Supplementary figures and images for: Large artery stiffening and mortality in a rat model of early vascular remodeling induced by intrauterine growth restriction and a high‐fat diet
Source: Physiol Rep. 2022 Dec 2;10(23):e15518. doi: 10.14814/phy2.15518 (PMC9718947; doi:10.14814/phy2.15518)

## Male

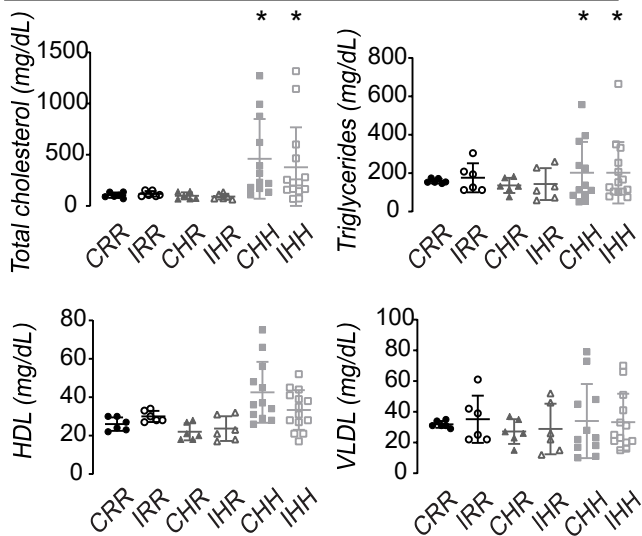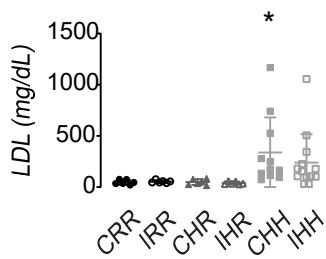

## Female

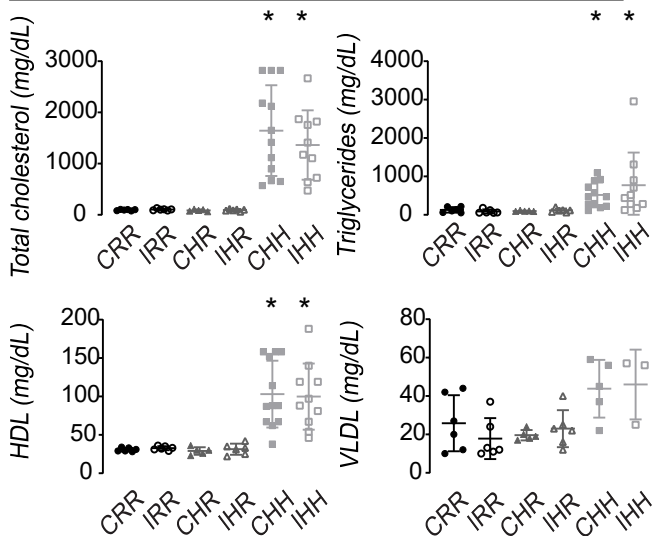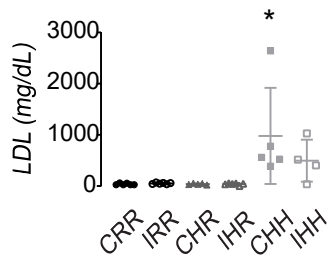

Supplement: Supplementary file 2 — Figure S1. [file PHY2-10-e15518-s003.pdf]

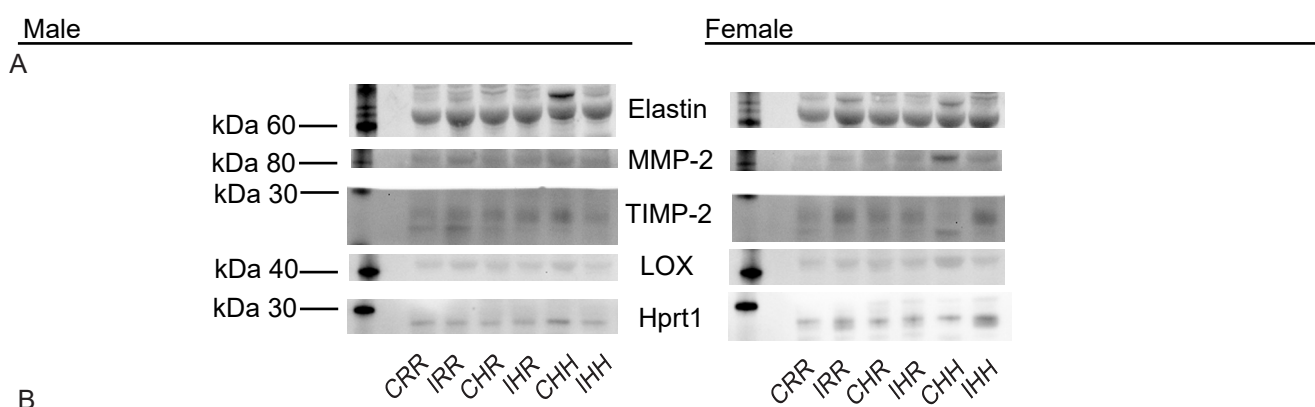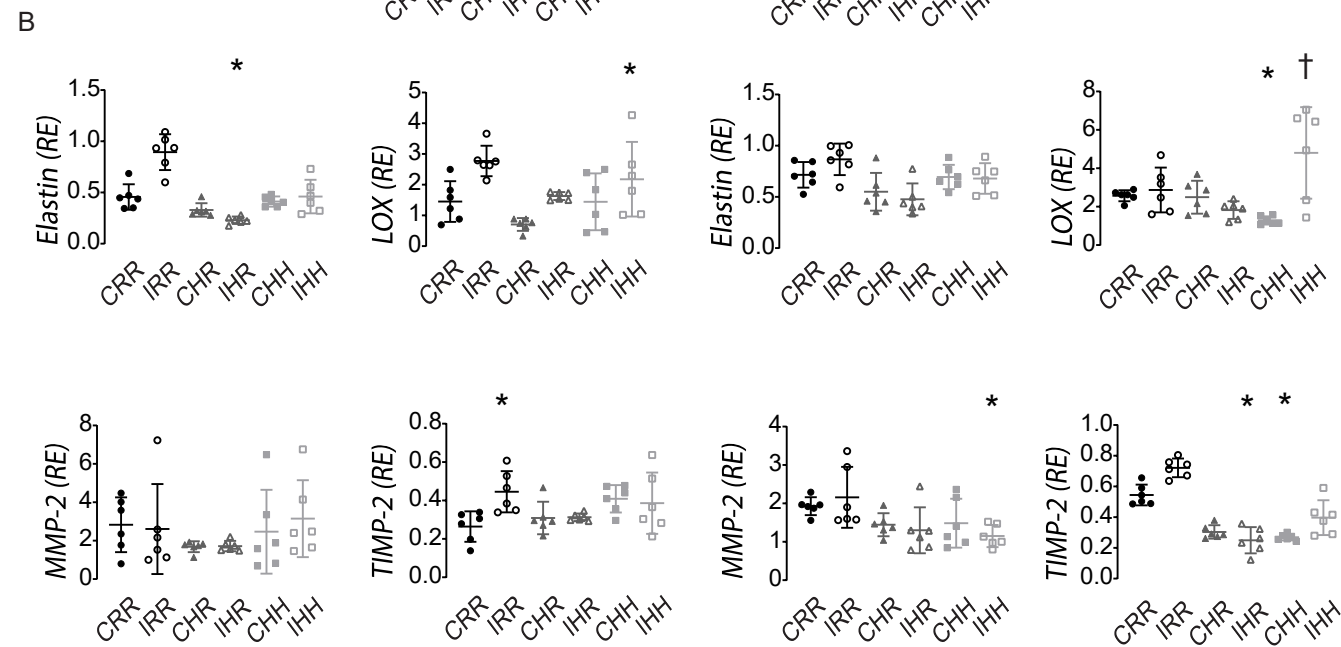

Supplement: Supplementary file 3 — Figure S2. [file PHY2-10-e15518-s002.pdf]

A

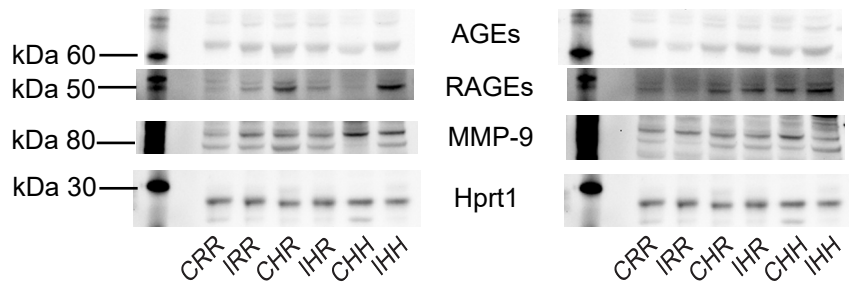

B

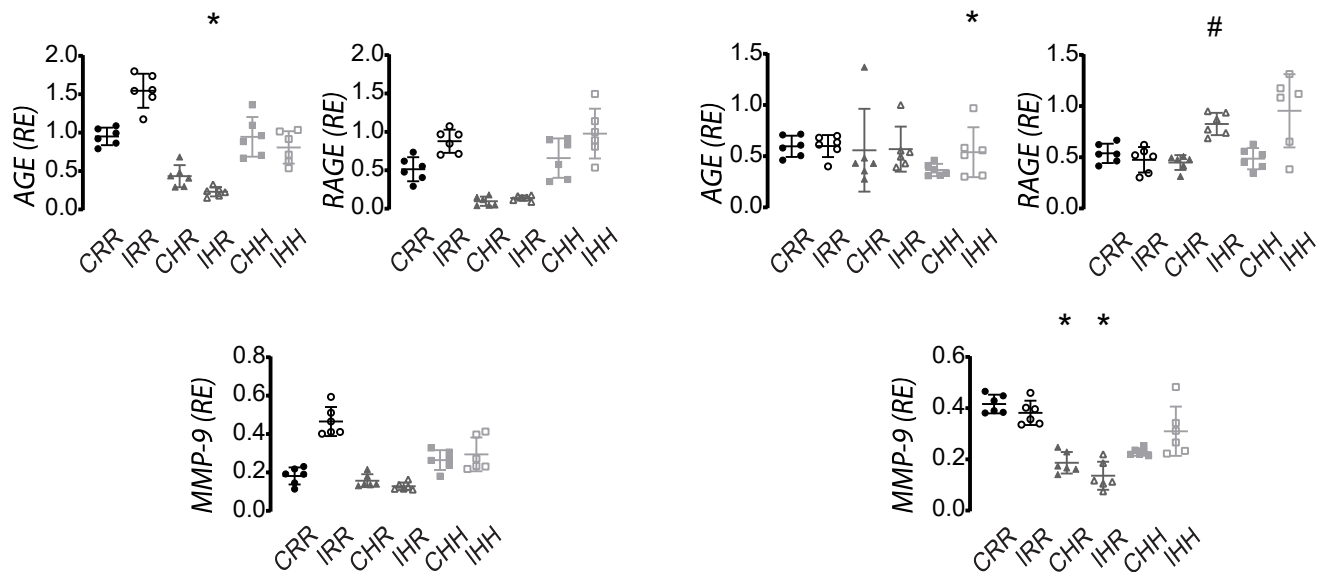

Supplement: Supplementary file 4 — Figure S3. [file PHY2-10-e15518-s005.pdf]

Male

Female

A

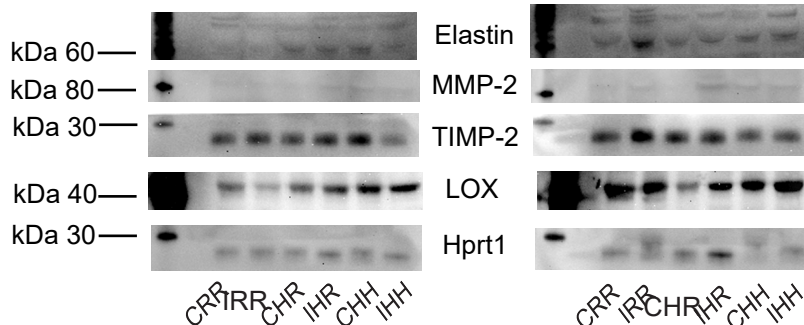

B

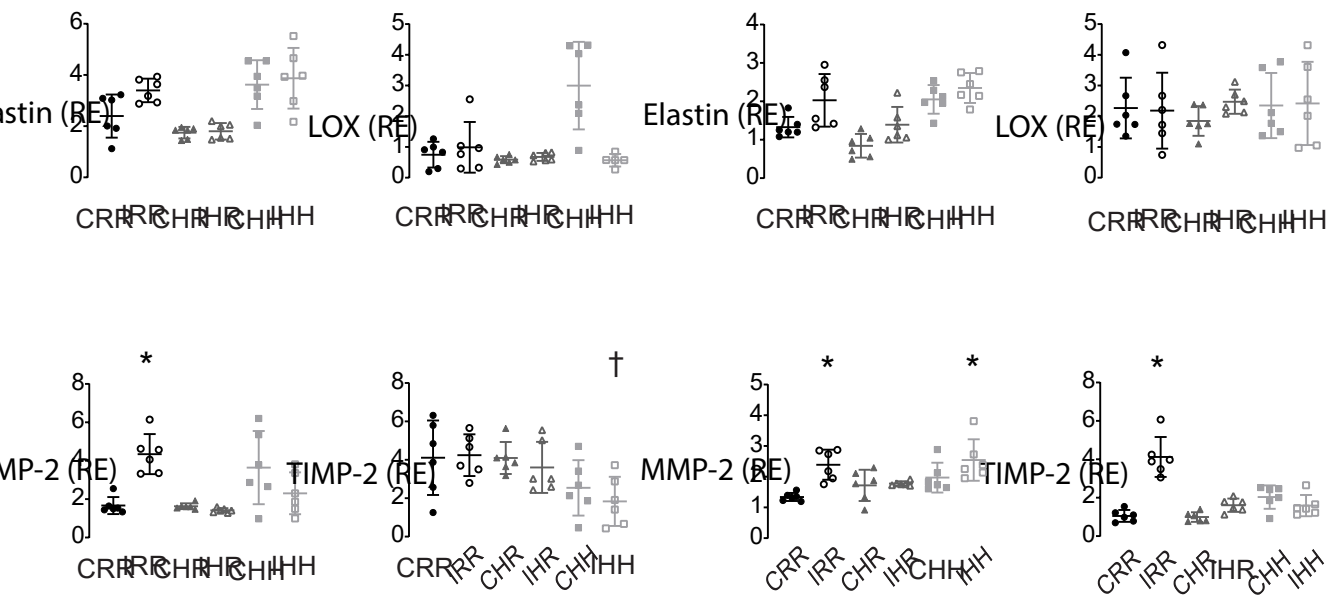

Supplement: Supplementary file 5 — Figure S4. [file PHY2-10-e15518-s001.pdf]

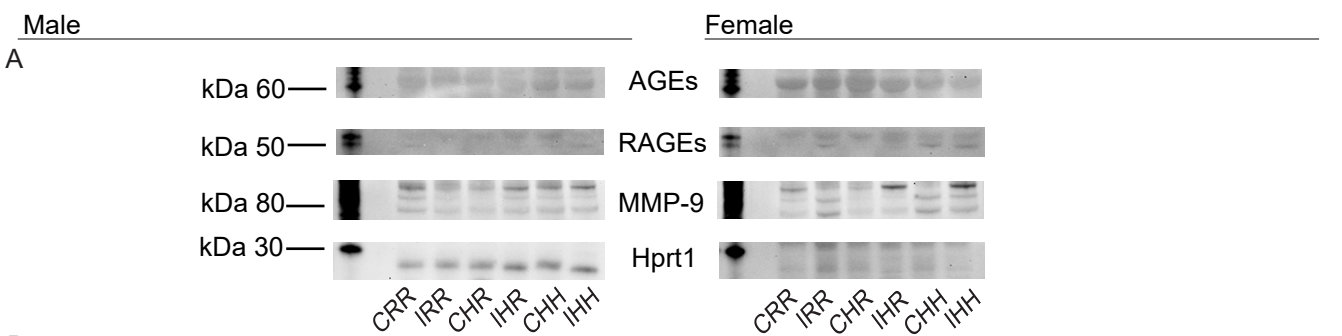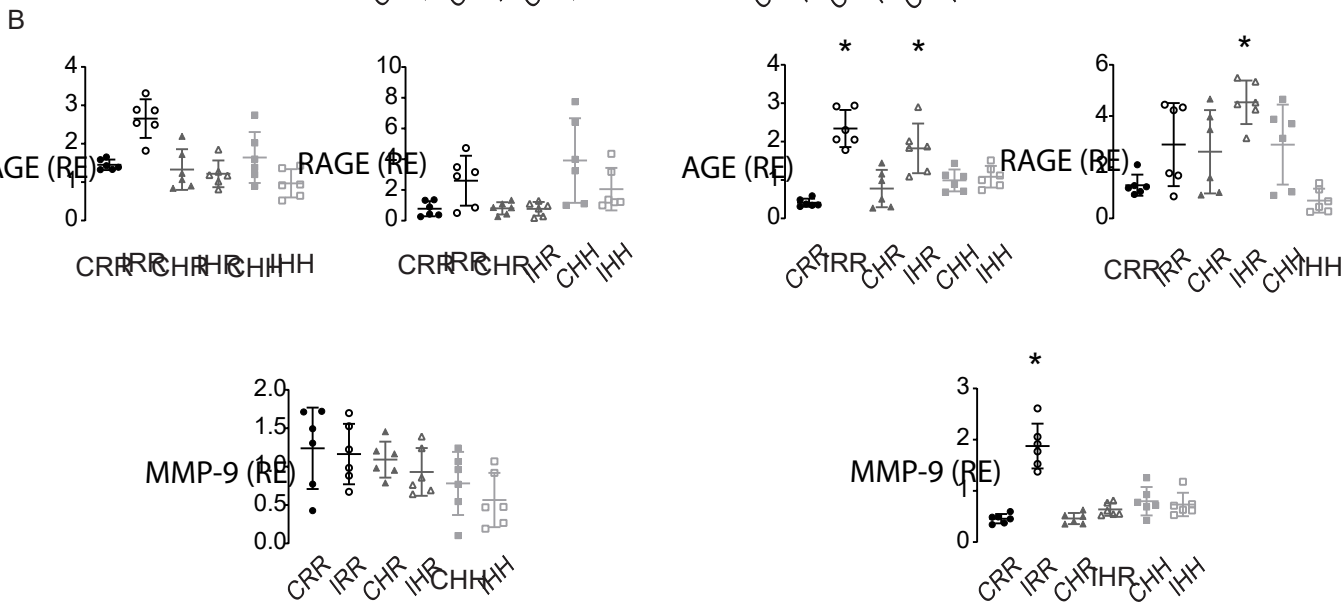

Supplement: Supplementary file 6 — Figure S5. [file PHY2-10-e15518-s004.pdf]
